# Delivery and neonatal outcomes of pregnant women during the Shanghai lockdown: A retrospective analysis

**DOI:** 10.3389/fped.2023.992908

**Published:** 2023-02-02

**Authors:** Fang-Yue Zhou, Cheng Li, Kai-Zhou Qin, Chuan Luo, He-Feng Huang, Yan-Ting Wu

**Affiliations:** ^1^ Obstetrics and Gynecology Hospital, Institute of Reproduction and Development, Fudan University, Shanghai, China; ^2^ International Peace Maternity and Child Health Hospital, School of Medicine, Shanghai Jiao Tong University, Shanghai, China; ^3^ Research Units of Embryo Original Diseases, Chinese Academy of Medical Sciences (No. 2019RU056), Shanghai, China

**Keywords:** COVID-19, lockdown, delivery outcomes, prelabor rupture of membranes (PROM), neonatal outcomes

## Abstract

**Objectives:**

Shanghai witnessed an unprecedented outbreak of COVID-19 and experienced a strict lockdown from March 28, 2022 to May 31, 2022. Most studies to date are on the first lockdown after the outbreak in December 2019. This study aimed to examine the impact of lockdown on delivery and neonatal outcomes among uninfected pregnant women in the new phase of the COVID-19 outbreak.

**Methods:**

A retrospective analysis was conducted in the Obstetrics and Gynecology Hospital of Fudan University. Pregnant women without COVID-19 who delivered from March 28, 2022 to May 31, 2022 (lockdown group) and the same period in 2021 (non-lockdown group) were recruited for this study. Logistic regression models and 1 : 1 propensity score matching (PSM) were used to assess the effect of lockdown on delivery outcomes.

**Results:**

A total of 2,962 patients were included in this study, 1,339 of whom were from the lockdown group. Compared with the non-lockdown group, pregnant women giving birth during lockdown had an increased risk of term prelabor rupture of membranes (TPROM) (aOR = 1.253, 95% CI: 1.026–1.530), and decreased risks of postpartum hemorrhage (PPH) (aOR = 0.362, 95% CI: 0.216–0.606) and fetal malformation (aOR = 0.309, 95% CI: 0.164–0.582). The risk of large for gestational age (LGA) (aOR = 0.802, 95% CI: 0.648–0.992) and rate of admission to the neonatal intensive care unit (NICU) (aOR = 0.722, 95% CI: 0.589–0.885) also significantly declined. After 1 : 1 PSM, the impact of lockdown on the risk of TPROM (aOR = 1.501, 95% CI: 1.083–2.080), PPH (aOR = 0.371, 95% CI: 0.211–0.654), fetal malformation (aOR = 0.332, 95% CI: 0.161–0.684), LGA (aOR = 0.749, 95% CI: 0.594–0.945) and rate of admission to the NICU (aOR = 0.700, 95% CI: 0.564–0.869) all remained. There were no other delivery or neonatal outcomes affected by the lockdown after the COVID-19 outbreak.

**Conclusion:**

This study indicated a significant increase in the risk of term PROM, significant decreases in the risk of PPH, fetal malformation and LGA, and a marked decline in the rate of admission to the NICU during Shanghai Lockdown.

## Introduction

It has been more than two years since coronavirus disease 2019 (COVID-19) first broke out in China and the world has witnessed several waves of COVID-19 caused by different variants of severe acute respiratory syndrome coronavirus 2 (SARS-CoV-2) (1). Currently, the Omicron BA.2 variant is dominant in at least 68 countries (2). The latest studies indicated that the BA.2 variant might be more transmissible than the original Omicron lineage, BA.1 (3, 4). BA.2 variant also presented less pathogenicity than the early SARS-CoV-2 strains (5). During the first outbreak in December 2019, Shanghai only experienced partial small-scale lockdown. However, this new outbreak of COVID-19 in Shanghai in 2022, which was mainly induced by the Omicron BA.2 variant, was aggressive, with far more people infected than the first epidemic. Therefore, the city was declared under lockdown at the end of March, 2022 (6). This was the first whole-city lockdown in Shanghai, with much stricter measures. Almost all residents were confined to their homes and banned from going out, except for those who provided medical necessities.

Many studies have investigated the perinatal outcomes of SARS-CoV-2 (+) pregnant women and infants. They found that the risk of PROM was much higher than that of virus-negative women (7, 8) and infants with COVID-19 were more likely to be admitted to the NICU (9, 10). But there were not many studies paying attention to the second effects of the COVID-19 outbreaks, such as lockdown. Lockdown is an important measure to prevent the spread of infection and mitigate the impact of the COVID-19 pandemic on public health. Lockdown may bring about good effects such as reduced exposure to infections and air pollution outdoors (11), which might reduce the chance of relevant air pollution induced pregnancy related complications (12). As to the bad effects, lockdown might result in increased anxiety and limited access to healthcare sectors, especially for pregnant women (13, 14). All of these may have important impacts on maternal and neonatal outcomes. Exploring the effects of lockdown can provide suggestions for public health measures to improve maternal and infant health. Several investigators have explored the effects of lockdown on the health of pregnant women and their infants who were SARS-CoV-2 negative, but inconsistent conclusions were drawn. Some studies demonstrated that lockdown decreased the rate of preterm birth (15–17), while others suggested that there was an increased risk of preterm birth for women under lockdown in their second trimester (18). Furthermore, a prospective observational study conducted in Nepal indicated that the rate of institutional stillbirth and neonatal mortality significantly increased during the national COVID-19 lockdown in Nepal (19). Philip et al. reported an unprecedented reduction in births of very low birthweight and extremely low birthweight infants during lockdown (20). In contrast, Charles et al. showed that very low birthweight and stillbirth rates were not significantly altered (21). However, the majority of the previously mentioned studies were conducted in the first year of the COVID-19 outbreak. Given the changes in transmissible rate, pathogenicity and public perception of SARS-CoV-2, Shanghai Lockdown offered us an opportunity to assess whether the whole-city lockdown had effects on pregnant women and their infants in the new phase of the COVID-19 outbreak.

Therefore, by paying attention to pregnant women giving birth during the Shanghai Lockdown, we aimed to evaluate the impact of lockdown on delivery and neonatal outcomes and provide evidence for strategies to improve maternal and infant health during the lockdown.

## Methods

### Study design and participants

This retrospective study was conducted at the Obstetrics and Gynecology Hospital of Fudan University, Shanghai, China, which is well-known in Shanghai and surrounding areas (including Jiangsu Province, Zhejiang Province and Anhui Province). Due to the Shanghai Lockdown officially beginning on March 28, 2022 (6) and ending on June1, 2022 (24), patients who had given birth with a gestational age >20 weeks at the Obstetrics and Gynecology Hospital of Fudan University from March 28, 2022 to May 31, 2022 were recruited in this study as the lockdown group. Considering that Shanghai was not greatly affected by the COVID-19 epidemic during the same period in 2021, we chose patients who gave birth at the same hospital from March 28, 2021 to May 31, 2021, as the non-lockdown group. According to the hospital policy, pregnant women have to undergo nucleic acid testing before they come to hospital for antenatal care, delivery or follow-up. All pregnant women included in this study were SARS-CoV-2 negative during pregnancy. And women are not suggested to receive Anti- SARS-CoV2 vaccine during pregnancy in China.

### Data collection

Data of all eligible populations were collected from the hospital electronic medical system, including basic demographic characteristics [age, age of husband, prepregnancy body mass index (BMI), insurance, marriage status, race, residence, education and occupation], reproductive history (parity, number of previous abortions, history of previous ectopic pregnancy, and mode of conception), lifestyle (history of smoking and drinking), pregnancy complications (number of fetuses, gestational hypertension disease, gestational diabetes mellitus (GDM), intrahepatic cholestasis of pregnancy (ICP), gestational thyroid dysfunction, placental abruption, abnormal placental position, and meconium-stained amniotic fluid (MSAF)), delivery outcomes (gestational age, mode of delivery, premature birth (PTB), prelabor rupture of membranes (PROM), postpartum hemorrhage (PPH) and fetal malformation), and neonatal outcomes [gender, birthweight, birthweight for gestational age, Apgar score within five minutes after birth and admission to neonatal intensive care unit (NICU)]. Height and prepregnancy weight were applied to obtain prepregnancy BMI, which was then categorized according to Chinese cutoff points (25). Gestational age was calculated based on the date of the last menstrual period. The mode of conception was classified according to whether the patient was undergoing assisted reproductive technology. The mode of delivery was divided into either vaginal or cesarean delivery.

### Diagnostic criteria

According to the International Classification of Diseases codes of discharge diagnosis, we obtained information about pregnancy complications and outcomes, including gestational hypertension disease, GDM, ICP, gestational thyroid dysfunction, placental abruption, abnormal placental position, MSAF, and PPH. PROM was defined as the rupture of membranes before the onset of labor (26). If PROM occurred before 37 weeks of gestation, the diagnosis of preterm PROM (PPROM) was made; otherwise, the diagnosis was term PROM (TPROM). PTB was defined as a birth prior to 37 gestational weeks (27). PTB could be divided into very early preterm birth (VPTB) and late preterm birth (LPTB) according to gestational age. VPTB referred to PTB occurring before 34 weeks of gestation, while LPTB was defined as a birth between 34 and 37 gestational weeks. According to whether PTB was spontaneous, PTB could also be categorized as either spontaneous PTB (S-PTB) or medically induced PTB (MI-PTB). Birthweight for gestational age was classified as small for gestational age (SGA), appropriate for gestational age (AGA), and large for gestational age (LGA). SGA was a child born with birthweight <10th centile for the gestational age and sex of the population. LGA was defined as birthweight >90th percentile for gestational age and sex. The reference data of SGA and LGA were from the newborn cross-sectional study of the INTERGROWTH-21st project (28).

### Statistical analysis

We first compared the baseline characteristics of the non-lockdown and lockdown groups, including demographic characteristics, pregnancy status, lifestyle, and pregnancy complications. Continuous variables are presented as medians and interquartile ranges. Categorical variables are presented as frequencies and percentages. The Mann–Whitney *U* test was employed for continuous variables with nonnormal distribution. The chi-square test or Fisher's exact test was applied to categorical variables. Next, we investigated the association between lockdown and delivery or neonatal outcomes by using logistic regression models to obtain crude and adjusted odds ratios (ORs) and corresponding 95% confidence intervals (CIs) (29). The potential confounders were determined by the significant differences in baseline characteristics between the non-lockdown and lockdown groups, including insurance, residence, marriage, occupation, and GDM.

In addition, to obtain similar baseline characteristics between the two groups, 1 : 1 propensity score matching (PSM) was performed. All of the participants in the lockdown group were matched, and a new non-lockdown group was generated. After matching, there was no significant difference between the two groups. Logistic regression models were used to explore the effects of lockdown on delivery or neonatal outcomes (29). Baseline characteristics with a *P* value < 0.2 were considered potential confounders. All analyses were conducted with SPSS version 25 (IBM SPSS Statistics, Chicago, IL, United States). All reported probability values were two-tailed, and the criterion for significance was set at a *P* value of <0.05.

## Results

### Sample baseline characteristics

A total of 2,962 pregnant women who gave birth at the Obstetrics and Gynecology Hospital of Fudan University were recruited for this retrospective study, 1,339 of whom were recruited from March 28, 2022, to May 31, 2022 (lockdown group), and 1,623 of whom were from the same period of 2021 and were treated as a control (non- lockdown group). The distribution of baseline characteristics, including demographic characteristics, pregnancy status, lifestyle, and pregnancy complications, in the unmatched samples and 1 : 1 PSM samples are depicted in Tables 1, 2. In the unmatched samples, compared with the non-lockdown group, women in the lockdown group were more likely to have medical insurance (86.56% vs. 83.55%, *P* = 0.020), get married (98.51% vs. 97.17%, *P* = 0.014), be local residents (89.02% vs. 83.36%, *P* < 0.001), take assisted reproductive technology (8.59% vs. 6.47%, *P* = 0.029), suffer from GDM (16.21% vs. 11.77%, *P* < 0.001), suffer from gestational hypertension (6.12% vs. 3.33%) and be less likely to be unemployed (2.09% vs. 12.26%) and have abortions (34.81% vs. 38.14%). Other characteristics were not significantly different between the two groups. After 1 : 1 PSM, 1,190 women in the lockdown group were matched with 1,190 women in the non-lockdown group, and the *P* values of all baseline characteristics were not significant.

**Table 1:** Baseline characteristics of unmatched samples and PSM samples.

	Unmatched Samples			1 : 1 PSM Samples		
	Non-lockdown	Lockdown	*P* value	Non-lockdown	Lockdown	*P* value
	(*N* = 1,623)	(*N* = 1,339)		(*N* = 1,190)	(*N* = 1,190)	
	No. (%)	No. (%)		No. (%)	No. (%)	
**Age, median (Q1-Q3), years**	31 (29–34)	32 (29–34)	0.075	31 (29–34)	32 (29–34)	0.330
**Age of husband, median (Q1-Q3), years**	32 (30–35)	32 (30–36)	0.131	32 (30–35)	32 (30–36)	0.404
**Pre-gestational BMI, median (Q1-Q3), kg/m^2^**	20.9 (19.2–22.8)	21.0 (19.4–23.0)	0.161	21.0 (19.3–22.8)	20.8 (19.3–22.9)	0.693
<18.5, kg/m2	231 (14.23)	196 (14.64)	0.072	167 (14.03)	173 (14.54)	0.819
18.5–23.9, kg/m2	1,160 (71.47)	906 (67.66)		854 (71.76)	835 (70.17)	
24.0–27.9, kg/m2	187 (11.52)	193 (14.41)		133(11.18)	146 (12.27)	
≥28.0, kg/m2	43 (2.65)	42 (3.14)		36 (3.03)	36 (3.03)	
**Insurance**			**0** **.** **020**			0.715
Yes	1,356 (83.55)	1,159 (86.56)		1,032 (86.72)	1,038 (87.23)	
No	267 (16.45)	180 (13.44)		158 (13.28)	152 (12.77)	
**Marriage**			**0** **.** **014**			0.722
Married	1,577 (97.17)	1,319 (98.51)		1,173 (98.57)	1,175 (98.74)	
Single	46 (2.83)	20 (1.49)		17 (1.43)	15 (1.26)	
**Race**						0.756
Han	1,593 (98.15)	1,316 (98.28)	0.789	1,168 (98.15)	1,170 (98.32)	
Others	30 (1.85)	23 (1.72)		22 (1.85)	20 (1.68)	
**Residence**			**<0** **.** **001**			0.115
Residents	1,353 (83.36)	1,192 (89.02)		1,083 (91.01)	1,060 (89.08)	
Immigrants	270 (16.64)	147 (10.98)		107 (8.99)	130 (10.92)	
**Education**			0.835			0.233
High school and below	169 (10.41)	146 (10.90)		101 (8.49)	116 (9.75)	
Bachelor	1,060 (65.31)	851 (63.55)		793 (66.64)	809 (67.98)	
Master and above	360 (22.18)	292 (21.81)		296 (24.87)	265 (22.27)	
**Occupation**			**<0** **.** **001**			0.957
Employed	1,266 (78.00)	1,279 (95.52)		1,131 (95.04)	1,132 (95.13)	
Self-employed	158 (9.74)	32 (2.39)		30 (2.52)	31 (2.61)	
Unemployed	199 (12.26)	28 (2.09)		29 (2.44)	27 (2.27)	
**Parity**			0.707			0.871
0	1,190 (73.32)	999 (74.61)		907 (76.22)	896 (75.29)	
1	390 (24.03)	306 (22.85)		254 (21.34)	264 (22.18)	
≥2	43 (2.65)	33 (2.46)		29 (2.44)	30 (2.52)	
**Number of previous abortions**			**0** **.** **026**			0.136
0	1,004 (61.86)	872 (65.12)		775 (65.13)	797 (66.97)	
1–2	559 (34.44)	403 (30.10)		379 (31.85)	344 (28.91)	
≥3	60 (3.70)	63 (4.71)		36 (3.03)	49 (4.12)	
**History of previous ectopic pregnancy**			0.974			0.737
No	1,590 (97.97)	1,312 (97.98)		1,171 (98.40)	1,173 (98.57)	
Yes	33 (2.03)	27 (2.02)		19 (1.60)	17 (1.43)	
**Assisted reproductive technology**			**0** **.** **029**			0.241
No	1,518 (93.53)	1,224 (91.41)		1,109 (93.19)	1,094 (91.93)	
Yes	105 (6.47)	115 (8.59)		81 (6.81)	96 (8.07)	

PSM, propensity score matching; BMI, body mass index.

**Table 2:** Pregnancy complications of unmatched samples and PSM samples.

	Unmatched Samples			1 : 1 PSM Samples		
	Non-lockdown	Lockdown	*P* value	Non-lockdown	Lockdown	*P* value
	(*N* = 1,623)	(*N* = 1,339)		(*N* = 1,190)	(*N* = 1,190)	
	No. (%)	No. (%)		No. (%)	No. (%)	
**Number of fetus**			0.106			0.612
Single birth	1,565 (94.43)	1,305 (97.46)		1,156 (97.14)	1,160 (97.48)	
Multiple birth	58 (3.57)	34 (2.54)		34 (2.86)	30 (2.52)	
**Gestational hypertension disease**			**0** **.** **002**			0.536
No	1,466 (90.33)	1,188 (88.72)		1,079 (90.67)	1,075 (90.34)	
Gestational hypertension	54 (3.33)	82 (6.12)		40 (3.36)	52 (4.37)	
Mild preeclampsia	75 (4.62)	52 (3.88)		51 (4.29)	47 (3.95)	
Severe preeclampsia or eclampsia	28 (1.73)	17 (1.27)		20 (1.68)	16 (1.34)	
**Gestational diabetes mellitus**			**<0** **.** **001**			0.509
No	1,432 (88.23)	1,122 (83.79)		1,035 (86.97)	1,024 (86.05)	
Yes	191 (11.77)	217 (16.21)		155 (13.03)	166 (13.95)	
**Pregestational diabetes mellitus**			0.394			0.220
No	1,599 (98.52)	1,324 (98.88)		1,170 (98.32)	1,177 (98.91)	
Yes	24 (1.48)	15 (1.12)		20 (1.68)	13 (1.09)	
**Intrahepatic cholestasis of pregnancy**			0.203			0.076
No	1,614 (99.45)	1,326 (99.03)		1,186 (99.66)	1,178 (98.99)	
Yes	9 (0.55)	13 (0.97)		4 (0.34)	12 (1.01)	
**Gestational thyroid dysfunction**			0.837			0.326
No	1,511 (93.10)	1,244 (92.91)		1,116 (93.78)	1,104 (92.77)	
Yes	112 (6.90)	95 (7.09)		74 (6.22)	86 (7.23)	
**Placental abruption**			0.822			0.803
No	1,612 (99.32)	1,331 (99.40)		1,181 (99.24)	1,183 (99.41)	
Yes	11 (0.68)	8 (0.60)		9 (0.76)	7 (0.59)	
**Abnormal placental position**			0.839			0.490
No	1,600 (98.58)	1,322 (98.73)		1,175 (98.74)	1,175 (98.74)	
Low-lying placenta	7 (0.43)	4 (0.30)		6 (0.50)	3 (0.0.25)	
Placenta previa	16 (0.99)	13 (0.97)		9 (0.0.76)	12 (1.01)	
**Meconium-stained amniotic fluid**			0.279			0.102
No	1,384 (85.27)	1,174 (87.68)		1,002 (84.20)	1,042 (87.56)	
Mild	44 (2.71)	31 (2.32)		37 (3.11)	27 (2.27)	
Moderate	69 (4.25)	51 (3.81)		52 (4.37)	45 (3.78)	
Severe	126 (7.76)	83 (6.20)		99 (8.32)	75 (6.30)	

PSM, propensity score matching.

### The effects of lockdown on delivery outcomes

We first conducted logistic regression models and then adjusted for all potential confounders to obtain crude and adjusted ORs in unmatched total samples (Table 3). We found that the risk of TPROM was increased by 25.3% (aOR=1.253, 95% CI: 1.026–1.530) during the lockdown time, while the risk of PPROM was similar (aOR = 1.261, 95% CI: 0.816–1.948). The risk of PPH declined (aOR = 0.362, 95% CI: 0.216–0.606). The rate of fetal malformation decreased more than 50% (aOR = 0.309, 95% CI: 0.164–0.582) in the lockdown group compared with the non-lockdown group. There were no differences between the two groups in the occurrence rates of PTB and cesarean sections. Logistic regression models were applied again in PSM samples (Table 4). After adjusting for confounding factors, the risk of TPROM remained significantly elevated (aOR = 1.253, 95% CI: 1.011–1.553). The risk of PPH (aOR = 0.371, 95% CI: 0.211–0.654) and fetal malformation (aOR = 0.332, 95% CI: 0.161–0.684) remained decreased.

**Table 3:** Delivery outcomes and neonatal outcomes of unmatched samples.

	Non-lockdown	Lockdown	OR (95% CI)	Adjusted OR (95% CI)
	(*N* = 1,623)	(*N* = 1,339)		
	No. (%)	No. (%)		
**Delivery outcomes**				
** Mode of delivery**				
Vaginal delivery	1,003 (61.80)	830 (61.99)	Reference	Reference
Cesarean delivery	620 (38.20)	509 (38.01)	0.992 (0.855–1.151)	0.955 (0.814–1.120)
** Premature rupture of membranes**				
No	1,317 (81.15)	1,035 (77.30)	Reference	Reference
TPROM	259 (15.96)	256 (19.12)	**1.258** **(1.039–1.522)**	**1.253** **(1.026–1.530)**
PPROM	47 (2.90)	48 (3.58)	1.300 (0.862–1.959)	1.261 (0.816–1.948)
** Premature birth**				
No	1,515 (93.35)	1,239 (92.53)	Reference	Reference
LPTB	85 (5.24)	77 (5.75)	1.108 (0.806–1.521)	1.176 (0.836–1.654)
VPTB	23 (1.42)	23 (1.72)	1.223 (0.683–2.190)	1.141 (0.62–2.094)
S-PTB	57 (3.51)	56 (4.18)	1.201 (0.825–1.750)	1.203 (0.811–1.784)
MI-PTB	51 (3.14)	44 (3.29)	1.055 (0.700–1.590)	1.115 (0.705–1.764)
** Postpartum hemorrhage**				
No	1,562 (96.24)	1,318 (98.43)	Reference	Reference
Yes	61 (3.76)	21 (1.57)	**0.408** **(0.247–0.674)**	**0.362** **(0.216–0.606)**
** Fetal malformation**				
No	1,573 (96.92)	1,326 (99.03)	Reference	Reference
Yes	50 (3.08	13 (0.97)	**0.308** **(0.167–0.570)**	**0.309** **(0.164–0.582)**
**Neonatal outcomes**				
** Gender**				
Male	830 (51.14)	693 (51.76)	Reference	Reference
Female	793 (48.86)	646 (48.24)	0.976 (0.44–1.128)	0.993 (0.854–1.155)
** Birthweight**				
Low birth weight (<2,500 g)	83 (5.11)	80 (5.97)	1.170 (0.853–1.606)	1.213 (0.866–1.700)
Normal birth weight (2,500–3,999 g)	1,440 (88.72)	1,186 (88.57)	Reference	Reference
Macrosomia (≥4,000 g)	100 (6.16)	73 (5.45)	0.886 (0.649–1.210)	0.975 (0.702–1.352)
** Birthweight for gestational age**				
SGA	50 (3.08)	40 (2.99)	0.929 (0.608–1.418)	0.939 (0.602–1.463)
AGA	1,298 (79.98)	1,118 (83.50)	Reference	Reference
LGA	275 (16.94)	181 (13.52)	**0.764** **(0.623–0.937)**	**0.802** **(0.648–0.992)**
** Apgar score within 5 min after birth**				
>7	1,599 (98.52)	1,327 (99.10)	Reference	Reference
≤7	24 (1.48)	12 (0.90)	0.602 (0.300–1.209)	0.734 (0.350–1.540)
** NICU**				
No	1,303 (80.28)	1,138 (84.99)	Reference	Reference
Yes	320 (19.72)	201 (15.01)	**0.719** **(0.593–0.873)**	**0.722** **(0.589–0.885)**

OR, odds ratios; CI, confidence intervals; TPROM, term prelabor rupture of membranes; PPROM, preterm prelabor rupture of membranes; LPTB, late preterm birth; VPTB, very early preterm birth; S-PTB, spontaneous preterm birth; MI-PTB, medically induced preterm birth; SGA, small for gestational age infant; AGA, appropriate for gestational age; LGA, large for gestational age infant; NICU, neonatal intensive care unit.

Adjusted confounders included insurance, residence, marriage, occupation, assisted reproductive technology, number of previous abortion, gestational diabetes mellitus and gestational hypertension disease.

**Table 4:** Delivery outcomes and neonatal outcomes of PSM samples.

	Non-lockdown	Lockdown	OR (95% CI)	Adjusted OR (95% CI)
	(*N* = 1,190)	(*N* = 1,190)		
	No. (%)	No. (%)		
**Delivery outcomes**				
** Mode of delivery**				
Vaginal delivery	726 (61.01)	745 (62.61)	Reference	Reference
Cesarean delivery	464 (38.99)	445 (37.39)	0.935 (0.792–1.103)	0.909 (0.768–1.076)
** Premature rupture of membranes**				
No	966 (81.18)	924 (77.65)	Reference	Reference
TPROM	188 (15.80)	227 (19.08)	**1.262** **(1.020–1.562)**	**1.253** **(1.011–1.553)**
PPROM	36 (3.03)	39 (3.28)	1.133 (0.714–1.797)	1.068 (0.670–1.701)
** Premature birth**				
No	1,113 (93.53)	1,105 (92.86)	Reference	Reference
LPTB	61 (5.13)	68 (5.71)	1.123 (0.787–1.602)	1.015 (0.707–1.457)
VPTB	16 (1.34)	17 (1.43)	1.070 (0.538–2.129)	1.062 (0.532–2.121)
S-PTB	40 (3.36)	50 (4.20)	1.259 (0.824–1.924)	1.202 (0.785–1.842)
MI-PTB	37 (3.11)	35 (2.94)	0.953 (0.596–1.524)	0.831 (0.513–1.344)
** Postpartum hemorrhage**				
No	1,145 (96.22)	1,173 (98.57)	Reference	Reference
Yes	45 (3.78)	17 (1.43)	**0.369** **(0.210–0.648)**	**0.371** **(0.211–0.654)**
** Fetal malformation**				
No	1,160 (97.48)	1,180 (99.16)	Reference	Reference
Yes	30 (2.52)	10 (0.84)	**0.328** **(0.159–0.673)**	**0.332** **(0.161–0.684)**
**Neonatal outcomes**				
** Gender**				
Male	616 (51.76)	616 (51.76)	Reference	Reference
Female	574 (48.24)	574 (48.24)	1.000 (0.851–1.174)	0.993 (0.845–1.168)
** Birthweight**				
Low birth weight (<2,500 g)	58 (4.87)	68 (5.71)	1.174 (0.818–1.683)	1.094 (0.760–1.575)
Normal birth weight (2,500–3,999 g)	1,063 (89.33)	1,062 (89.24)	Reference	Reference
Macrosomia (≥4,000 g)	69 (5.80)	60 (5.04)	0.870 (0.610–1.243)	0.871 (0.609–1.245)
** Birthweight for gestational age**				
SGA	35 (2.94)	335 (28.15)	0.959 (0.595–1.545)	0.940 (0.581–1.519)
AGA	964 (81.01)	1,005 (84.45)	Reference	Reference
LGA	191 (16.05)	150 (12.61)	**0.753** **(0.598–0.949)**	**0.749** **(0.594–0.945)**
** Apgar score within 5 min after birth**				
>7	1,178 (98.99)	1,179 (99.08)	Reference	Reference
≤7	12 (1.01)	11 (0.92)	0.916 (0.403–2.084)	0.877 (0.385–2.002)
** NICU**				
No	954 (80.17)	1,011 (84.96)	Reference	Reference
Yes	236 (19.83)	179 (15.04)	**0.716** **(0.578–0.886)**	**0.700** **(0.564–0.869)**

PSM, propensity score matching; OR, odds ratios; CI, confidence intervals; TPROM, term prelabor rupture of membranes; PPROM, preterm prelabor rupture of membranes; LPTB, late preterm birth; VPTB, very early preterm birth; S-PTB, spontaneous preterm birth; MI-PTB, medically induced preterm birth; SGA, small for gestational age infant; AGA, appropriate for gestational age; LGA, large for gestational age infant; NICU, neonatal intensive care unit.

Adjusted confounders included residence, number of previous abortion, intrahepatic cholestasis of pregnancy and meconium-stained amniotic fluid.

### The effects of lockdown on neonatal outcomes

The same procedures of analysis were carried out to explore the differences in neonatal outcomes during the Shanghai Lockdown. The results of unmatched total samples indicated that the risk of LGA and admission to the NICU were both decreased in the lockdown group in contrast with the non-lockdown group, with an aOR of 0.802 (95% CI: 0.648–0.992) and 0.722 (95% CI: 0.589–0.885), respectively, after adjusting for confounders (Table 3). After 1 : 1 PSM, the risks of LGA (aOR = 0.749, 95% CI: 0.594–0.945) and admission to the NICU (aOR = 0.700, 95% CI: 0.564–0.869) still declined during the lockdown. The other variables, including neonatal gender, birthweight, and Apgar score, were similar between the two groups (Table 4).

## Discussion

In this retrospective study, we discovered that women giving birth at the Obstetrics and Gynecology Hospital of Fudan University during the period of Shanghai Lockdown were more likely to be local residents and have medical insurance. The risk of term prelabor rupture of membranes was significantly increased, while the rate of admission to the neonatal intensive care unit was decreased in the lockdown group compared with the non-lockdown group.

Several studies have investigated the effects of the COVID-19 outbreak on perinatal outcomes (30). Kugelman et al. evaluated the effect of the COVID-19 epidemic on changes in the obstetrical emergency department profile and did not limit them to women suffering from COVID-19. They found that higher proportions of women presented with PROM during the epidemic (31). Du et al. reported that the risk of PROM for pregnant women who were SARS-CoV-2 negative was significantly increased, with an OR of 1.11, during the first seven months of the COVID-19 outbreak (32). Our results also indicated that exposure to the lockdown resulted in an increased risk of PROM, especially term PROM. Most studies agreed that the COVID-19 pandemic and lockdown had adverse effects on maternal mental health, such as distress and depression (14, 22). Maternal anxiety and depression might lead to a higher prevalence of PROM (33). PPROM can result from a list of pathologic mechanisms, and infection has been suggested to be one of the most important inducements (26). It is possible that lockdown did not affect the rate of exposure to various pathogens, so the incidence of PPROM was not significantly changed. Although term PROM may result from a normal physiologic weakening of the membranes combined with shearing forces created by uterine contractions, it can induce intrauterine infections such as chorioamnionitis and endometritis (26). Geng et al. demonstrated that lockdown prolonged the waiting time at home after the occurrence of term PROM, which was associated with increasing maternal C-reactive protein (34). These findings suggest that service provision, such as the timely transport of women with term PROM, should be guaranteed during lockdown, and measures to prevent infection after PROM need to be popularized to pregnant women. In our study, there was no difference in the PTB rate between the two groups. This was in accordance with the results of our team's previous study, which discovered that the PTB rate of pregnant women was stable when lockdown was carried out in their third trimester (18). However, for women get pregnant during the first outbreak of COVID-19, restriction measures were associated with a reduced rate of preterm birth before 34 weeks (11). It suggests that different phases of pregnancy affected by COVID-19 lockdown may result in different perinatal outcomes. In addition, the risks of PPH and fetal malformation declined. This phenomenon could be partly attributed to selection bias caused by the traffic restriction between different districts during the lockdown. Before the lockdown, pregnant women with higher risks of PPH or fetal malformation referred to give birth in the Obstetrics and Gynecology Hospital of Fudan University. It is one of the most famous and highest-level obstetrics and gynecology hospitals in Shanghai. However, due to the traffic restriction between different districts in Shanghai, pregnant women with higher risk of PPH or fetal malformation were not able to go to our hospital if they did not live in the same district.

Regarding the impact of lockdown on neonatal outcomes, our study revealed that the rate of admission to the NICU was markedly reduced. However, the conclusions in other studies were not accordant. A systematic review and meta-analysis found no significant effects of the COVID-19 epidemic on NICU admission (23). The studies conducted in Northern Ghana (44% vs. 56%) and Japan (adjusted incidence rate ratio = 0.76) both suggested a decrease in admission to the NICU during the COVID-19 outbreak (35, 36). The decline may be attributed to the following reasons. First, the provision of the NICU was insufficient because many medical staff were quarantined home during the lockdown period. Second, the criteria for admission to the NICU were more stringent as a result of limited medical resources and increased unwillingness of parents to be separated from their babies during the outbreak. Third, movement restriction and more attention to infection prevention measures may reduce the rates of intrauterine infections and alleviate maternal physical stress (36). Fourth, inconvenient mobility between different districts may lead to selection bias. This finding indicates that more attention should be given to how to structure and optimize teamwork in the NICU. The strategy of adopting or modifying family-centered care (FCC) during the COVID-19 pandemic and lockdown may be beneficial to relieving parental anxiety and stress (37). In addition, the risk of LGA declined. It was possibly correlated with dietary changes such as fewer chances of eating at restaurants during the lockdown period. In a web-based survey during the COVID-19 pandemic lockdown in Italy, 44.3% pregnant women reported eating in a healthier way. This survey also reported lockdown reduced physical exercise (38). It was hard to evaluate the extent to which diet and exercise habits have changed. Besides, inadequate supplies of food in the early time of lockdown and anxiety could also affect maternal diets and weight gain.

This study presents the effects of Shanghai Lockdown on delivery and neonatal outcomes in the new phase of the COVID-19 outbreak. We applied different statistical models (logistic regression models and PSM) to evaluate the association between lockdown and delivery or neonatal outcomes. There were also some limitations in our study. As a retrospective analysis, some confounders, such as incomes, maternal mental health and dietary and physical exercise habits, were not measured. In addition, it was a single-center study with a limited sample size; thus, selection bias was inevitable. Maternal censuses with large sample sizes are warranted in the future to confirm the findings in our study.

## Conclusion

This study examined the impact of the Shanghai Lockdown on delivery and neonatal outcomes. Our findings indicated a significant increase in the risk of term PROM, significant decreases in the risk of PPH, fetal malformation, and LGA, and a marked decline in the rate of admission to the NICU during lockdown compared with those in the same period of 2021, when Shanghai was not under COVID-19 blockade. These results suggested that timely medical service provision, education programs, improved collaboration in the NICU and strategies of FCC may be helpful for maternal and child health care during lockdown resulted from infectious diseases.

## Data Availability

The raw data supporting the conclusions of this article will be made available by the authors, without undue reservation.
